# High-Temperature Response Polylactic Acid Composites by Tuning Double-Percolated Structures

**DOI:** 10.3390/polym15010138

**Published:** 2022-12-28

**Authors:** Haiwei Yao, Rong Xue, Chouxuan Wang, Chengzhi Chen, Xin Xie, Pengfei Zhang, Zhongguo Zhao, Yapeng Li

**Affiliations:** 1 Textile and Clothing, College of Chemical Engineering, Shaanxi Polytechnic Institute, Xianyang 712000, China; 2 National and Local Engineering Laboratory for Slag Comprehensive Utilization and Environment Technology, School of Materials Science and Engineering, Shaanxi University of Technology, Hanzhong 723000, China; 3 School of Textile Science and Engineering, Xi’an Polytechnic University, Xi’an 710048, China

**Keywords:** crystallization, electrical conductivity, temperature response behavior, positive temperature coefficient

## Abstract

Due to the properties of a positive temperature coefficient (PTC) effect and a negative temperature coefficient (NTC) effect, electrically conductive polymer composites (CPCs) have been widely used in polymer thermistors. A dual percolated conductive microstructure was prepared by introducing the polybutylene adipate terephthalate phase (PBAT) into graphene nanoplatelets (GNPs)-filled polylactic acid (PLA) composites, intending to develop a favorable and stable PTC material. To achieve this strategy, GNPs were selectively distributed in the PBAT phase by injection molding. In this study, we investigated the crystallization behavior, electrical conductivity, and temperature response of GNP-filled PLA/PBAT composites. The introduction of GNPs into PLA significantly increased PLA crystallization capacity, where the crystallization onset temperature (T_o_) is raised from 116.7 °C to 134.7 °C, and the crystallization half-time (t_1/2_) decreases from 35.8 min to 27.3 min. The addition of 5 wt% PBAT increases the electrical conductivity of PLA/PBAT/GNPs composites by almost two orders of magnitude when compared to PLA/GNPs counterparts. The temperature of the heat treatment is also found to play a role in affecting the electrical conductivity of PLA-based composites. Increasing crystallinity is favorable for increasing electrical conductivity. PLA/PBAT/GNPs composites also show a significant positive temperature coefficient, which is reflected in the temperature–electrical resistance cycling tests.

## 1. Introduction

In recent years, due to the high conductivity, lightweight properties, corrosion resistance, and good processability of electrically conductive polymer composites (CPCs) [1,2,3], they have been widely used in electromagnetic shielding materials (EMI) [4,5,6], sensors [7,8], capacitors [9,10], and other fields [11,12,13]. Due to the non-degradable properties of petroleum-based polymers, bio-based polymers have been extensively studied, and CPCs prepared with degradable polymers as a matrix have become a research hotspot [14,15,16,17]. CPCs have a positive temperature coefficient (PTC) of CPCs, that is, the resistance of the composite increases with temperature, and the negative temperature coefficient (NTC) of CPCs, the resistance of the composite, decreases with an increase in temperature. These two important temperature-related characteristics have great theoretical significance for application in self-protection fuses and temperature-sensitive sensors [18,19,20,21,22]. However, CPCs are usually accompanied by two effects of PTC and NTC in a certain temperature range, which limits their application. Therefore, testing the temperature range of the PTC and NTC effects of CPCs has an important application value [23]. Moreover, there is a growing demand to design a CPC material possessing tunable PTC characteristics.

Up to now, research on regulating the PTC characteristics of CPCs has been reported [24,25,26]. It is known that the increasing temperature can induce the expansion of the polymer matrix and break the conductive links, exhibiting the PTC effect, whereas the re-agglomeration of the conductive additives can induce the NTC effect [27]. Most of the research published mainly focuses on the changes in temperature region in the vicinity of the melting point, thus, the PTC is considered an advantage to broaden the application area. Dai et al. [24] employed a segregated and double-percolated composite microstructure to develop a favorable NTC material by selectively distributing graphene in a polyamide 6 (PA6) phase between isolated ultra-high molecular weight polyethylene (UHMWPE) particles, achieving a relatively linear NTC effect through the whole heating process. Liu et al. [25] prepared high-density polyethylene (HDPE)/carbon fiber (CF) and isotactic polypropylene (iPP)/CF composites by melt blending, and only observed an abnormal PTC effect in an extremely narrow temperature range. Wang et al. [26] prepared a prototype of a multi-walled carbon nanotube (MWCNT)/epoxy resin flexible sensor, which shows obvious PTC and NTC phenomena. Nevertheless, the aforementioned methods were only feasible with their certain microstructure or chemical composition; importantly, most of the research mainly focuses on the one-component polymer, which would also lead to poor PTC reproducibility. It is still a challenge to tune the PTC efficiently.

Due to the outstanding characteristics of graphene nanoplatelets, such as a large aspect ratio and high electrical conductivity, the temperature response behaviors of CPCs filled with graphene attracted enormous attention in recent years. Pang et al. [27] constructed the segregated structure by controlling the migration of GNPs to the surface of ultra-high molecular weight polyethylene (UHMWPE), resulting in an increasing PTC intensity. Thus, the interaction of conductive fillers plays a pivotal role in the evolution of the conductive network in the temperature field. Although numerous research studies focused on investigating the morphology, electronic, and mechanical properties of the GNPs/polylactic acid (PLA) composites [28,29,30], the temperature response behaviors of the GNPs/PLA composites have rarely been studied so far. In particular, PLA as the biodegradable material combined with conductive fillers for temperature sensors is expected to have a potential multipurpose application in bio-nanomaterials and eco-friendly functional materials field [17,31].

In this paper, PLA/GNPs composites were prepared by an injection molding machine, and the internal conductive network structure of PLA/GNPs composites was adjusted by adding PBAT to form a double percolation conductive network structure inside the polymer. In addition, we explored the change in electrical conductivity of GNPs/PLA composites with time under different heat treatment temperatures, and the temperature-sensitive performance of PLA/GNPs composite in the temperature range of 37~140 °C was studied. At the same time, the influence of PBAT on the crystallization, electrical conductivity, and temperature sensitivity of PLA/PBAT/GNPs composites was systematically explored.

## 2. Materials and Methods

### 2.1. Materials

Polylactic acid (PLA), brand name 4032D, produced by Nature works; polybutylene adipate terephthalate (PBAT), brand name C1200, produced by BASF, Germany; graphene nanoplatelets (GNPs), thickness 4~20 nm, diameter 5~10 μm, Chengdu Institute of Organic Chemistry, Chinese Academy of Sciences.

### 2.2. Sample Preparation

Pure PLA pellets (100 g) were first blended with various contents of GNPs (0, 1, 2, 2.5, 3, 3.5, 4, and 4.5 wt%) and PBAT (5, 10, 20, 30, 40, and 50 wt%) using a twin-screw extruder (SHJ-20, Nanjing Haisi Extrusion Equipment Co., Ltd., Nanjing, China). The screw speed was 80 rpm and the temperature profile from hopper to die was from 160 to 195 °C. Then, the injection molding machine (HTF90W1, Ningbo Haitian Plastic Machine Group Co. LTD, Ningbo, China) was used to prepare standard dumbbell samples at an injection temperature profile of 190 to 200 °C from the hopper to the nozzle to simplify the name of the composites; PLAGNP_x_PB_y_ denotes the composite GNPs weight content as x and as PB weight content as y. For example, PLAGNP_3.5_PB_10_ composite contains 3.5 wt% GNPs and 10 wt% PBAT.

### 2.3. Characterization

#### 2.3.1. Scanning Electron Microscopy (SEM)

To explore the dispersion state of GNPS in PLA and PBAT/PLA, the spline was first quenched using liquid nitrogen and then the section was observed using scanning electron microscopy (SEM). Scanning electron microscope (SEM) model: JSM-6390LV, Rigaku, Tokyo, Japan.

#### 2.3.2. Differential Scanning Calorimetry (DSC)

To explore the influence of adding GNPs on the crystallinity of the composite, the samples were tested by DSC (model instrumentation: TGA/DSC1, Mettler-Toledo Instruments, Zurich, Switzerland). First, samples weighing 5~10 mg were placed in the crucible and were heated from 40 to 200 °C at 10 °C/min under a nitrogen atmosphere. They were then kept at 200 °C for 5 min to remove thermal history. Finally, the temperature was cooled from 200 to 40 °C at 3 °C/min.

The relative crystallinity ($X_{c}$) can be expressed by Equation (1) [32]:

(1) $$X_{c}=\frac{{\Delta H}_{m}-{\Delta H}_{cc}}{{\Delta H}_{f}\omega_{PLA}}\times 100\%$$

where ${\Delta H}_{m}$ is the melting enthalpy of PLA, ${\Delta H}_{cc}$ is the cold crystallization enthalpy of PLA, and ${\Delta H}_{f}$ is standard melting enthalpy of 100%, being 93 J/g; $\omega_{PLA}$ is the mass fraction of PLA in the composite material.

#### 2.3.3. Conductivity Testing

The electrical conductivity of samples was tested using an isolation resistance meter (TA2684A, Changzhou Tonghui Electronics, Changzhou, China).

The electrical conductivity (σ) for each sample was calculated [33]:

(2) σ=L/RS

where σ is the conductivity of the material, R is the volume resistance, and S is the cross-sectional area.

#### 2.3.4. Temperature Response Behavior Analyses

**Figure 1:**
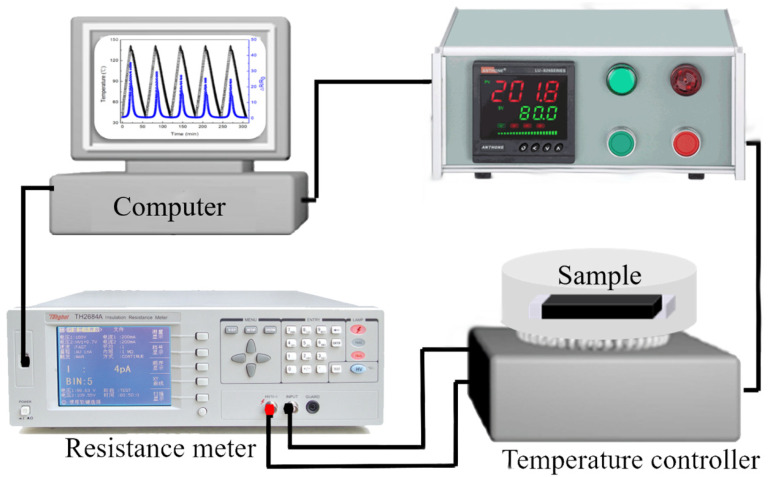
The self-designed equipment schematic diagram.

The temperature response behavior of PLA/PBAT/CNTs was investigated by self-designed equipment using an isolation resistance meter and temperature controller (WCY-SJ, Nanjing Sangli Electronic Equipment Factory, Nanjing, China). Based on the crystalline temperature of PLAGNP_3_._5,_ the testing temperature was set from 25 to 100, 120 and 135 °C at 3 °C/min. In the meantime, the resistance changes of samples were online tested, as shown in Figure 1.

## 3. Results and Discussion

### 3.1. Electrical Conductivity of CPCs

**Figure 2:**
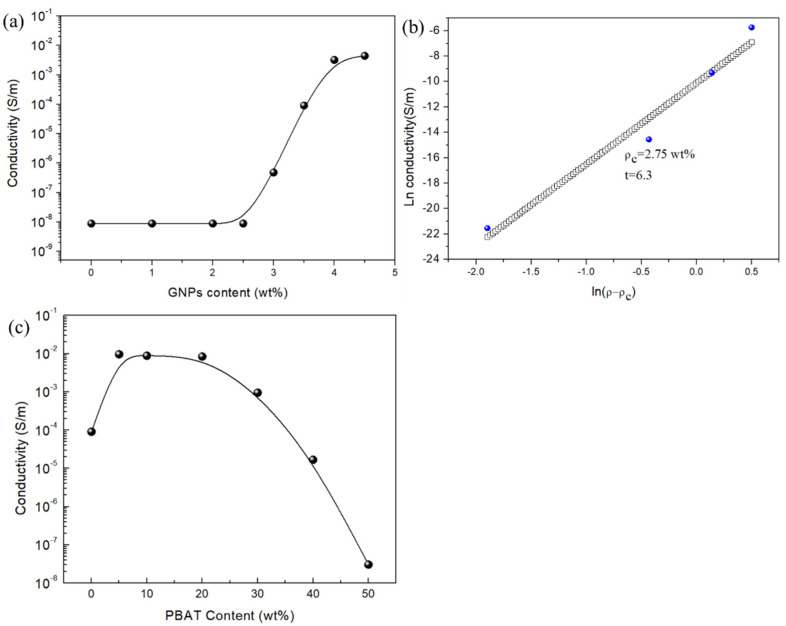
(**a**) Electric conductivity of PLA/GNPs composite, (**b**) the fitting curves of electric conductivity, and (**c**) electric conductivity curve of PLA/PBAT/GNPs composite as a function of PBAT content.

To explore the influence of GNPs and PBAT addition on the electrical conductivity of PLA/PBAT/GNPs composites, all samples were tested as shown in Figure 2. Figure 2a shows that when GNPs content is in the range between 0 and 2.5 wt%, there is no change in the electrical conductivity of PLA/GNPs samples, showing the insulating behavior (<$10^{-8}$ S/m). The reason for this is that GNPs are not connected to form a conducting path in the PLA matrix, so electron transmission depends primarily on electrons hopping from adjacent fillers. With further increasing the GNPs content, the conductivity of the composites gradually increases. When the GNPs content exceeds 3 wt%, the conductivity of the samples increases rapidly from a value of 4.7 × $10^{-7}$ to 3.3 × $10^{-3}$ S/m, indicating that the internal conducting path of the composites is starting to form. Furthermore, when the GNPs content is greater than 4 wt%, the conductivity of the samples has no obvious change, showing that the inner conducting network of the composite is perfect, and the composite material is gradually converted into a conductor. This indicates that the filler content plays a vital role in constructing a conductive network path for the conductivity of composites. For comparison, the classical percolation theory shown in Equation (3) was cited to predict the conductive percolation value of the composite material in which the fitting value was 2.75 wt% [33].

(3) $$\sigma \left(p\right)=\delta {\left(p-p_{c}\right)}^{t}$$

where σ(p) is the conductivity of the composite material, δ is the conductivity of the filler, p is the content of the conductive filler, $p_{c}$ is the percolation threshold of the composite, and *t* is the critical index.

According to the above analysis, the GNPs content of 3.5 wt% was chosen to explore the influence of PBAT content on the electric conductivity, as shown in Figure 2c. From Figure 2c, it can be seen that the curve of the PLAGNP_3.5_PB_y_ shows an “n” type change trend. When 5 wt% PBAT is added, the conductivity of the composite rises to 9.6 × $10^{-3}$ S/m, which is two orders of magnitude higher than that of PLAGNP_3.5_. This is due to the low content of PBAT playing a repulsive role in the composite material, which promotes a part of GNPs migration from the PLA phase to the interface of PBAT and forms the conductive paths. When the amount of PBAT added is further increased from 5 to 20 wt%, the conductivity of PLAGNP_3.5_PB_y_ shows a trend of first increasing and then maintaining a plateau. This is due to the migration of GNPs into the PBAT phase and a small amount of PBAT is not enough to agglomerate GNPs in a large amount. Therefore, the process of increasing PBAT content is the process of increasing GNPs migration capacity. However, with increasing PBAT content to 50 wt%, the conductivity of the composite drops to 3.04 × $10^{-8}$ S/m, which is about three orders of magnitude lower than that of PLAGNP_3.5_. This is due to the higher melting viscosity of PBAT and a large number of GNPs migrating into the PBAT phase, resulting in the large-area agglomeration and no more conductive paths.

### 3.2. The Phase Morphology Analysis of CPCs

**Figure 3:**
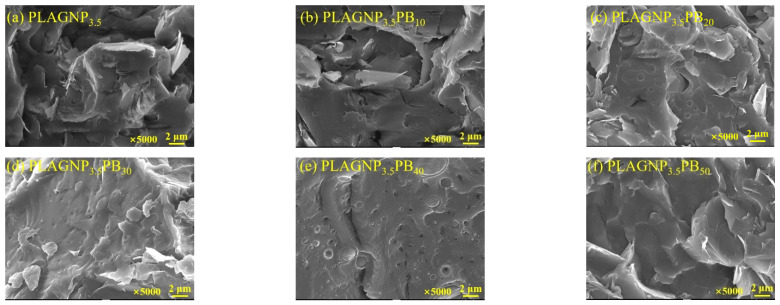
SEM images of PLA/PBAT/GNPs composites.

To better explain the effect of phase structure on the electrical conductivity of PLA/PBAT/GNPs composites, SEM was employed, as shown in Figure 3. As can be seen in Figure 3a, GNPs are distributed either flatly or vertically in the PLA matrix, and the surface structure of PLA is roughened, indicating that a large number of GNPs are overlapping on the surface of the quenched section. This phenomenon causes the PLA/GNPs composites to have a higher percolation threshold. With introducing PBAT into PLA/GNPs composites, a large number of white dots (sea-island structure) appear on the surface, which indicates low compatibility between PLA and PBAT. Moreover, the GNPs can be discerned on the surface. However, when further increasing the PBAT content from 30 to 40 wt%, the size of the dispersed phase is enlarged and the GNPs also vanish. When the PBAT content is increased to 50 wt%, the dispersed phase vanishes and forms co-continuous structures [14]. Thus, with increasing PBAT content, the changes in electric conductivity can be explained: (1) the high PBAT melting viscosity can induce the agglomeration of GNPs, thereby decreasing the formation of the conductive network; (2) it is easier for GNPs to migrate from the PLA phase to the PBAT phase due to the intermolecular forces [34], thus, adding high PBAT content into PLA matrix can destroy the conductive network and reduce the electrical conductivity.

### 3.3. The Crystalline Properties of CPCs

**Figure 4:**
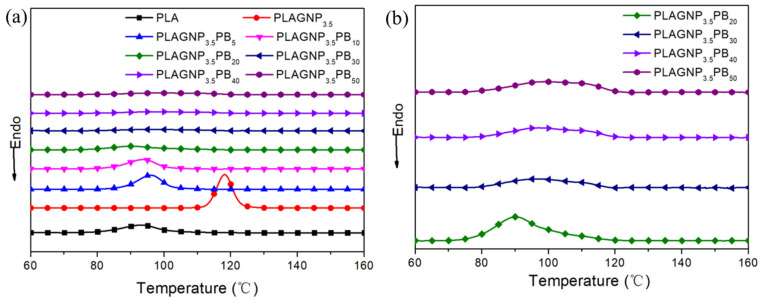
(**a**) DSC curve of PLAGNP_3.5_PB_y_ composite and (**b**) the magnification curve diagram.

To explore the influence of the addition of GNPs and PBAT on the crystallization properties of CPCs, a study of non-isothermal behaviors of CPCs was carried out. The results are shown in Figure 4. Table 1 shows the onset crystallization temperature (T_0_), the peak crystallization temperature (T_p_), and the half-time of crystallization (T_1/2_). It can be seen from Figure 3a that the addition of GNPs shifts the T_0_ and T_p_ of the composites to high temperature, in which the T_0_ increases from 116.7 to 134.7 °C, and T_p_ increases from 93.2 to 120 °C. Moreover, introducing GNPs into the PLA matrix also significantly decreases T_1/2_ from 35.8 to 27.3 min. These phenomena demonstrate that GNPs with a large surface can provide more nucleation sites and promote the crystalline process. However, introducing PBAT into PLA/GNPs composites displays an inverse phenomenon. When adding PBAT into PLA/GNPs composites, the T_0_ and T_p_ decrease, while T_1/2_ increases from 27.3 min to 36 min, as shown in Figure 4 and Table 1. The addition of PBAT results in the packaging of a large number of GNPs into PBAT, thereby reducing the number of nucleation sites and hindering the motion of PLA chains, which greatly inhibits the process of crystal growth.

### 3.4. The Isothermal Temperature Response of CPCs

**Figure 5:**
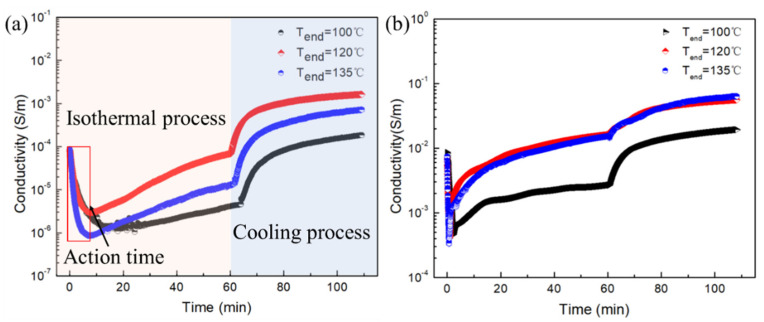
The temperature response behavior of PLAGNP_3.5_ (**a**) and PLAGNP_3.5_PB_20_ composites (**b**).

Based on the above analysis, 20 wt% PBAT was chosen to explore the temperature response behaviors of CPCs. An online isothermal process was performed and the data are shown in Figure 5, in which the samples were treated at varying temperatures (T_end_ = 100, 120, and 135 °C). As shown in Figure 4a, the conductivity of PLAGNP_3.5_ first decreases from 6.99 × $10^{-5}$ S/m to 1.37 ×$10^{-6}$ S/m with an action time of 14.15 min and then gradually increases. Moreover, by increasing the heat-treated temperature from 100 to 135 °C, the action time is reduced to 7.77 min (seen in Table 2), indicating that the increase in heat treatment temperature can enhance the action time. Afterward, the electric conductivity shows a similar increase trend, in which with time passing, the electrical conductivity gradually increases. The reason for this is that during the isothermal process, the crystalline structure that appears within the polymer chains is progressively refined, and the “crystal repulsion” effect results in a large displacement of GNPs into the amorphous region, leading to a slow increase in electrical conductivity. At the end of the cooling process after 60 min, the conductivity initially rises sharply and then gradually stabilizes due to the rapid growth of the crystal at the beginning of the cooling step, which makes the repulsion effect of the crystal most obvious. It also can be seen from Figure 5b that during the subsequent isothermal process at different isothermal temperatures, the conductivity of PLAGNP_3.5_PB_20_ also tends to slowly increase. However, compared to the PLAGNP_3.5_ composite, the conductivity of the PLAGNP_3.5_PB_20_ drops from 8.62 × $10^{-3}$ S/m to 4.60 × $10^{-4}$ S/m at 100 °C and action time is significantly shortened to 2.19 min.

**Figure 6:**
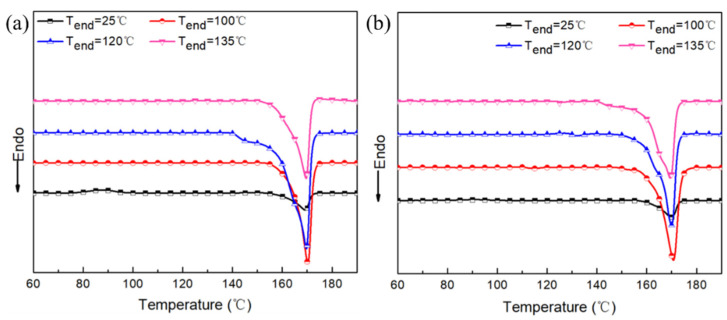
(**a**) The heating DSC curve of PLAGNP_3.5_ composite and (**b**) DSC curve of PLAGNP_3.5_PB_20_ composite at different heat treatment temperatures.

The corresponding heat-treated samples were further tested by DSC to explore the crystallinity changes of PLA-based composites, as shown in Figure 6 and the related parameters are shown in Table 2. Figure 6 shows that PLAGNP_3.5_ and PLAGNP_3.5_PB_20_ have similar changing trends, in which increasing the T_end_ can enhance the melting enthalpy and melting temperature, indicating that the crystalline structures become more perfect. Moreover, the relative crystallinity of samples, as shown in Table 2, is the highest when T_end_ is 120 °C, corresponding to 52.3% (PLAGNP_3.5_) and 46.8% (PLAGNP_3.5_PB_20_), respectively. This is in agreement with the final conductivity of the composites when the isothermal temperature is 120 °C in the time plot of the electrical conductivity, which proves the effects of “crystalline rejection” on the conductivity of the composites.

### 3.5. The Non-Isothermal Temperature-Response Behaviors of CPCs

**Figure 7:**
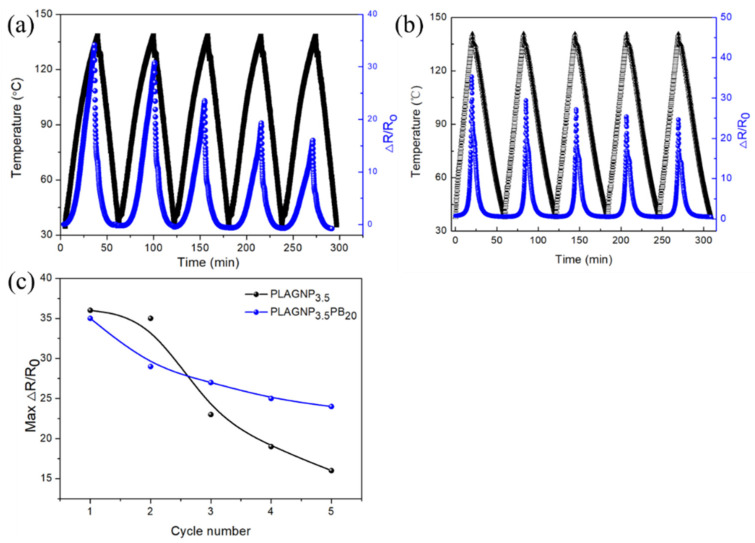
The electrical conductivity versus temperature curves of (**a**) PLAGNP_3.5_, (**b**) PLAGNP_3.5_PB_20_ and (**c**) the changes of max ΔR/R_0_.

To explore the non-isothermal temperature response behavior of PLAGNP_3.5_ and PLAGNP_3.5_PB_20_, non-isothermal temperature tests were performed and shown in Figure 7. The ratio ΔR/R_0_ (ΔR = R − R_0_, R is the real resistance and R_0_ is the initial resistance) represents the resistance changes with the changing temperature, in which the maximum ΔR/R_0_ was used to characterize the PTC intensity. As can be seen from Figure 6a, when increasing the testing temperature from room temperature to 135 °C, the value of ΔR/R_0_ is also increased, demonstrating the PTC phenomenon under the single cycle process. Moreover, the maximum ΔR/R_0_ of the PLAGNP_3.5_ composites (seen Figure 7c) show a gradual decrease with increasing cycle times. The reason for this is that PLA is an amorphous polymer when the temperature is increased and decreased for the PLAGNP_3.5_ composite, and the temperature field, therefore, destroys some of the imperfect crystalline structure in the composites; so, in the slow cooling process, the imperfect crystal gradually achieves perfect crystallization so that the effect of “crystal repulsion” is increased, and the maximum ΔR/R_0_ is reduced in the following cycle. Furthermore, introducing the 20 wt% PBAT into PLA/GNPs composites has no obvious changes on the PTC phenomenon. However, the PTC value is enhanced and becomes more stable after the first cycle process compared with PLAGNP_3.5_, in which the PTC value can be kept at 25. Thus, introducing PBAT into PLA/GNPs composites can provide a new method to prepare the temperature sensor with more sensibility and stability.

## 4. Conclusions

In this study, the PLA/GNPs/PBAT composites were prepared by injection molding and the electric conductivity, crystallization performance, and temperature response behavior were investigated in detail. Increasing GNPs content can gradually enhance the electrical conductivity and the percolation threshold is 2.75 wt% by fitting with a classic percolation theory. Moreover, the crystallization performance of PLAGNP_3.5_ is improved by the addition of GNPs, which has a stronger ability to induce the crystallization of PLA due to their strong nucleation effect, including growth in crystallization rate and crystallinity. As for PLA/PBAT/GNPs composites, introducing a lower PBAT content can form a better conductive network, further improving the electrical conductivity, while decreasing crystalline temperatures, due to the high melting viscosity of PBAT and migration process of GNPs from PLA to PBAT. During the temperature-response testing, introducing PBAT can significantly shorten the action time from 14.15 min to 2.19 min and the cycle temperature-response stability is also gradually improved, in which the PTC value can be kept at 25 when compared with PLAGNP_3.5_. The investigation of the microstructure evolution of a conductive network provides a guideline for the design and fabrication of temperature-sensing devices with high conductivity, high stability, and high-temperature sensitivity in a variety of applications.

## Figures and Tables

**Table 1:** Comparison table of the crystallization process of PLAGNP_3.5_PB_y_ composite.

Sample	T_0_ (°C)	T_p_ (°C)	T_1/2_ (min)	Sample	T_0_ (°C)	T_p_ (°C)	T_1/2_ (min)
PLA	116.7	93.2	35.8	PLAGNP_3.5_PB_20_	120.9	90.3	36.0
PLAGNP_3.5_	130.8	118.2	27.3	PLAGNP_3.5_PB_30_	120.6	97.6	34.1
PLAGNP_3.5_PB_5_	112.9	96.0	34.8	PLAGNP_3.5_PB_40_	125.3	97.2	33.5
PLAGNP_3.5_PB_10_	110.7	93.8	35.7	PLAGNP_3.5_PB_50_	125.3	98.8	33.2

**Table 2:** The relative conductive parameters and DSC data at different heat treatment temperatures.

Heat Treatment Temperature	Initial Conductivity (10^−5^ S/m)		Minimum Conductivity (10^−6^ S/m)		Action Time (min)		Relative Crystallinity (%)	
	a	B	a	b	a	b	a	b
T_end_ = 25 °C							27.4	32.5
T_end_ = 100 °C	6.99	862	1.37	460	14.15	2.19	44.3	35.0
T_end_ = 120 °C	8.42	711	2.97	410	7.25	1.00	52.3	46.8
T_end_ = 135 °C	8.72	700	8.61	332	7.77	0.69	50.3	46.0

a represents PLAGNP_3.5_ and b represents PLAGNP_3.5_PB_20_.

## Data Availability

The data presented in this study are available on request from the corresponding author.
